# 

**Published:** 1898-04

**Authors:** 


### Communicated.

---

### AMERICAN MEDICAL ASSOCIATION.

---

DENVER, MARCH 7TH, 1898.

GENTLEMEN :—The American Medical Association will hold its fifty-first annual meeting in the city of Denver, Colorado, June 7, 8, 9 and 10th, 1898. During the same week the Colorado State Medical Society will convene, as will also the Rocky Mountain Interstate Medical Association, whose membership embraces the physicians of Arizona, Colorado, Idaho, Montana, New Mexico, Utah and Wyoming,

As is customary in connection with these meetings there will be an Exhibit. For this purpose the Gettysburg Building has been secured. It is especially well adapted and conveniently located to all hotels and section meeting places. In the same building will also be located: Office for registration, Bureau of Information, Post Office and Office for endorsing Railroad certificates.

It is the intention of the profession in Denver to make this meeting surpass all previous meetings of the Association and already the indications point to a large attendance.

The physicians throughout the country begin to understand the meaning of western hospitality and will take advantage of the special concessions to be made by the railroads to visit Colorado. The exhibitors will also have the opportunity of coming in contact with the doctors of a new region who may not again be so easily and generally reached. They are men who are open to new ideas and able to appreciate them.

Address all communications regarding exhibits to

C. K. FLEMING, Chairman, California Bldg.

### To Readers and Correspondents.

Communications intended for publication, and exchange journals and papers, should be addressed to the Editor of The American Journal of Dental Science, No. 845 N. Eutaw St. Baltimore, Md. All letters relating to the business of the Journal, or containing cash remittances, to be directed to Snowden & Cowman Mfg. Co., 9 W. Fayette Street, Baltimore, Md. Articles for publication should be received by the Editor at least two weeks previous to the first of the month on which the number in which it is designed for them to appear is issued.

☞ The American Journal of Dental Science will contain fifty pages of reading matter in each number, making a volume of about Six Hundred pages,

EXCLUSIVE OF ADVERTISEMENTS.

